# The American Dental Trade Association

**Published:** 1889-07

**Authors:** 


### Editorial.

---

### THE AMERICAN DENTAL TRADE ASSOCIATION.

For some time past we had been intending to prepare an editorial on the operations of the American Dental Trade Association, and did so for the June issue of the Journal, but it was crowded out in the "clean up" preparatory to a change in printers. In it we said "that, after investigating the plan of organization and practical working of the Association, we were fully convinced that, instead of its being a detriment, it had been a real benefit to the profession, since, by its influence, better business methods had been established: a better feeling and fairer dealing exist now between its members than previously. The constant reduction in the price of burs, socket instruments, and gas shows that it is in no sense a trust or monopoly." We have come in contact with the members of the Association the past year, and have been afforded every opportunity to observe the operation of the rules governing the Association, and, so far as we can judge, they are fair and just as between men engaged in the same business. There is only one clause in the Articles of Association that has any appearance of being exclusive, and that is the one relating to new members. This can be, and has been, sometimes so used as to prevent the admission of good men who desired to enter the retail business. If this clause was liberally interpreted there would be no reason to condemn the action of the Association.

While we say this much in favor of the Association as a body, we cannot help admitting that there is a wide-spread feeling in the profession regarding certain averred abuses connected with the business of manufacturing dental supplies. This feeling has to a great extent been misdirected in the past, but the publication of the explanation will tend to set the profession aright in the matter.

We therefore gladly give space to the circular, hoping that it may have the desired effect, and correct any misunderstanding regarding the purposes and aim of the Association. We cannot, however, help thinking that it would have been better had this been done when the organization was first accomplished, for its publication at the present time may have a different effect from that expected by some members of the Association, by directing the attention of the profession from the main body to individual members, and may thus be the means of raising questions that may be rather difficult to answer. Although abuses do exist, their correction does not lie in attacking the American Dental Trade Association.

